# Serum angiopoietin-1 concentration does not distinguish patients with ischaemic stroke from those presenting to hospital with ischaemic stroke mimics

**DOI:** 10.1186/s12872-022-02918-w

**Published:** 2022-11-04

**Authors:** Joseph V. Moxon, Ann-Katrin Kraeuter, James Phie, Sheryl Juliano, Georgina Anderson, Glenys Standley, Cindy Sealey, Richard P. White, Jonathan Golledge

**Affiliations:** 1 Queensland Research Centre for Peripheral Vascular Disease, College of Medicine and Dentistry, James Cook University, Townsville, QLD 4811 Australia; 2 Centre for Molecular Therapeutics, Australian Institute of Tropical Health and Medicine, James Cook University, Townsville, QLD 4811 Australia; 3 Faculty of Health and Life Sciences, , Northumbria University, PsychologyNewcastle Upon Tyne, UK; 4 Brain, Performance and Nutrition Research Centre, Northumbria University, Newcastle Upon Tyne, UK; 5 NUTRAN, Northumbria University, Newcastle Upon Tyne, UK; 6 Department of Neurology, Townsville University Hospital, Townsville, QLD 4811 Australia; 7 Department of Vascular and Endovascular Surgery, Townsville University Hospital, Townsville, QLD 4811 Australia

**Keywords:** Angiopoietin, Stroke, Biomarker

## Abstract

**Background:**

A previous study found that circulating angiopoietin-1 (angpt-1) concentrations were significantly lower in patients who had a recent ischaemic stroke compared to healthy controls. The primary aim of this study was to assess whether serum angpt-1 could be used as a diagnostic test of ischemic stroke in patients presenting to hospital as an emergency. Exploratory analyses investigated the association of proteins functionally related to angpt-1 (angpt-2, Tie-2, matrix metalloproteinase-9 and vascular endothelial growth factors A, C and D) with ischaemic stroke diagnosis.

**Methods:**

Patients presenting to Townsville University Hospital for emergency assessment of stroke-like symptoms were consecutively recruited and provided a blood sample. After assessment by a consultant neurologist, patients were grouped into those who did, or did not have ischaemic stroke. The potential for serum angpt-1 to diagnose ischaemic stroke was assessed using receiver operator characteristic (ROC) curves. Cross-sectional analyses appraised inter-group differences in the serum concentration of other proteins.

**Results:**

One-hundred and twenty-six patients presenting to Townsville University Hospital for emergency assessment of stroke-like symptoms were recruited (median time from symptom onset to hospital presentation: 2.6 (inter-quartile range: 1.2–4.6) hours). Serum angpt-1 had poor ability to diagnose ischaemic stroke in analyses using the whole cohort, or in sensitivity analyses (area under the ROC curve 0.51 (95% CI: 0.41–0.62) and 0.52 (95% CI: 0.39–0.64), respectively). No associations of serum angpt-1 concentration with ischaemic stroke severity, symptom duration or aetiology were observed. Serum concentrations of the other assessed proteins did not differ between patient groups.

**Conclusions:**

Serum angpt-1 concentration is unlikely to be useful for emergency diagnosis of ischaemic stroke.

**Supplementary Information:**

The online version contains supplementary material available at 10.1186/s12872-022-02918-w.

## Introduction

Global Burden of Disease data reveals that approximately 12 million incident stroke events occurred in 2019, ~ 60% of which were ischaemic [1]. Chemical thrombolysis using recombinant tissue plasminogen activator is used to treat ischaemic stroke but has a limited window of efficacy and is associated with a significant risk of bleeding complications [2–4]. Current guidelines recommend that ischaemic stroke diagnosis be confirmed through brain imaging and specialist assessment prior to instigating treatment [5] however this introduces geographical inequities in access to care [3, 6], and risk of misdiagnosis [7, 8], within a time critical window for intervention. In contrast acute myocardial infarction can be rapidly diagnosed and treated using objective serological data to guide clinical decision making [9, 10]. The discovery of similarly effective blood markers to diagnose ischaemic stroke has potential to streamline current management processes and improve patient outcome [8, 11, 12].

Animal studies suggest that the expression of the protein angiopoietin-1 (angpt-1) alters following an ischemic stroke, and that upregulating angpt-1 reduces the severity of cerebral infarction [4, 13]. A case–control study (336 cases and 321 healthy controls) also reported that median plasma angpt-1 concentration was threefold lower in patients who had a recent ischemic stroke than controls, indicating strong diagnostic potential (area under receiver operator characteristic curve was 0.95 (95% CI 0.93, 0.96)) [14]. To be clinically useful, an effective diagnostic blood test would need to differentiate between ischaemic stroke and other conditions presenting with similar symptoms. It is therefore vital to examine the ability for blood angpt-1 concentration to distinguish patients experiencing ischaemic stroke from those with other acute health problems mimicking stroke. The primary aim of this study was to assess whether serum angpt-1 concentrations could be valuable as a diagnostic test of ischemic stroke in consecutively recruited patients presenting as an emergency to hospital with a possible diagnosis of stroke. Exploratory analyses assessed the difference in serum concentrations of proteins functionally related to angpt-1 between patients with or without ischaemic stroke to identify whether they had additional biomarker potential.

## Materials and methods

### Patients

The current study consecutively recruited participants presenting to the Townsville University Hospital, Queensland, Australia, for investigation of stroke-like symptoms from 2017–2020. This study was conducted with approval from the Human Research Ethics Committees at James Cook University and the Townsville University Hospital, in accordance with the Declaration of Helsinki. For inclusion, patients had to be aged > 18 years and present to hospital within 24 h of symptom onset. Written informed consent was collected from all patients, or via a responsible 3^rd^ party if the patient was unable to provide consent in person. Patients consented via a third party were contacted and re-enrolled if they later regained the ability to consent in person. The study is reported in line with the Standards for Reporting Diagnostic accuracy studies (STARD) recommendations (2015) [15].

### Outcome assessment

**Fig. 1:**
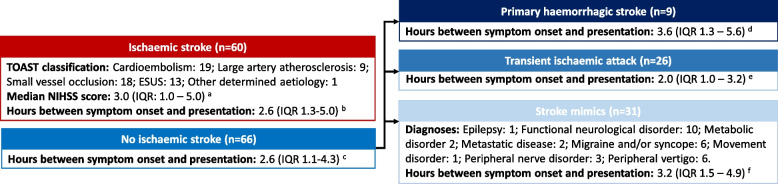
Patient flow for the current study; **a** Based on 44 observations (73.3% of group); **b** Based on 55 observations (91.7% of group); **c** Based on 57 observations (86.4% of group); **d** Based on 7 observations (77.8% of group); **e** Based on 25 observations (96.2% of group); **f** Based on 25 observations (80.6% of group). ESUS: Embolic Stroke of Undetermined Source

All diagnosis were made by a consultant neurologist (RW) blinded to the biomarker results. The primary outcome was ischaemic stroke diagnosis, based upon a clear clinical presentation and congruent brain imaging evidence of cerebral infarction (either computed tomography (CT) and/or magnetic resonance imaging conducted as part of standard care), in line with current guidelines [5]. Estimations of ischaemic stroke severity at presentation reported by attending neurologists during standard care were extracted from patient charts. Heterogeneity in the choice of stroke severity assessment tool was observed. The National Institutes of Health Stroke Severity Score (NIHSS) was the most consistently applied tool (used in approximately three quarters of recruited participants) and was therefore employed in the current study. Numbers of patients for whom this detail is missing are reported (Fig. 1). Ischaemic stroke aetiology was categorised according to the Trial of ORG 10,172 in Acute Stroke Treatment (TOAST) criteria [16] based on imaging and clinical findings for all recruited participants. Primary haemorrhagic stroke was diagnosed based on the presence of a hyper-dense area on cerebral CT [5]. Transient ischaemic attack was diagnosed based on stroke-like symptoms which resolved within 24 h of onset and no evidence of cerebral infarction on radiological imaging [5]. Stroke mimics were defined as a non-vascular condition presenting with acute or subacute stroke-like neurological deficits without brain infarction.

### Clinical risk factors and medications

Characteristics collected for each participant included sex, age, history of hypertension, diabetes mellitus coronary heart disease (CHD) and prescribed medications as previously described [17, 18]. Hypertension and diabetes were defined by a history of diagnosis or treatment for these conditions. CHD status was defined by a history of angina, myocardial infarction or coronary revascularization. Details of medications prescribed at the time of presentation were recorded.

### Blood samples & analysis

Peripheral blood samples were collected from all participants at recruitment as part of standard care and serum was processed by the Townsville University Hospital Pathology Department (Pathology Queensland) prior to storing at − 80 °C for later analysis. Serum angpt-1, matrix-metalloproteinase-9 (MMP-9) and Tie-2 concentrations were measured using commercially available ELISAs according to manufacturer’s directions (DANG 10, DMP900 and DTE200, respectively, R&D Systems, Minneapolis, USA). Serum angiopoietin-2 (angpt-2), and vascular endothelial growth factor (VEGF)-A, -C and -D concentrations were measured using the MilliPLEX MAP Human Angiogenesis/Growth Factor Magnetic Bead Panel—Cancer Multiplex Assay kit (HAGP1MAG-12 K, Merck, Australia), using the MagPIX platform, and analyses were conducted by a researcher blinded to patient diagnosis. Samples in which the assessed biomarker fell outside of the detectable limits of the test were excluded from analysis (detailed in Supplement 1). Biomarker analysis was conducted independently of patient care and had no potential to influence clinical outcome.

### Statistical analysis

Continuous variables were non-normally distributed (Shapiro–Wilk test) and are presented as median and inter-quartile range. Some variables (e.g. NIHSS score and time from symptom onset to hospital presentation) were not available for all patients and were not imputed owing to their non-random distribution within the population. Inter-group comparisons were performed using the Mann–Whitney U test, or Kruskall-Wallis test. Nominal data are presented as count and percent and were compared between groups using the Chi-squared test. Correlations between continuous variables (including serum protein concentrations) were tested using Spearman’s correlation. For all analyses a *p* value of < 0.05 were considered statistically significant. The ability of serum angpt-1 concentration to diagnose ischaemic stroke was assessed using receiver operator characteristic (ROC) curves (boot-strapped for 2000 iterations). Area under the ROC curve and 95% confidence intervals are reported. The initial analysis included all recruited participants, leading to a sensitivity analysis comparing patients with ischaemic stroke those with stroke mimics.

All analyses were performed using RStudio (Version 3.5.5) using the “car”, “dplyr”, “knitr”, “ggplot2″, “pROC”, “ggpubr”, “magrittr”, “tibble”, “ggsignif”,”ggsci” and”rlang” packages.

### Sample size calculation

The study was powered to assess the ability of serum angpt-1 concentration to diagnose ischaemic stroke as a primary outcome. Our previous case–control study reported that circulating angpt-1 had high potential to distinguish patients with acute ischaemic stroke from healthy controls evidenced by an area under the ROC curve of 0.946 (95% CI: 0.923–0.963) [14]. The current study utilized consecutively recruited participants suspected of suffering ischaemic stroke, and it was hypothesized that mechanisms underpinning neurological symptoms in the non-stroke patients may influence circulating angpt-1 concentrations. Accordingly, the current study was powered to test the ability of serum angpt-1 concentration to diagnose stroke with a conservative area under the ROC curve of at least 0.800, corresponding to ‘good’ clinical performance [19]. Assuming a 1:1 ischaemic:non-ischaemic stroke ratio and a 2-tailed alpha of 0.05, power calculations suggested that a minimum of 17 participants per group were needed to detect this with 90% power.

## Results

### Patient characteristics

A total of 126 participants were included in this study, 60 of whom were diagnosed with ischaemic strokes (Table 1, Fig. 1). Ischaemic strokes within the cohort predominantly arose due to cardioembolism or small vessel occlusion, accounting for 31.7% and 30.0% of ischaemic stroke presentations, respectively (Fig. 1). Median (inter-quartile range) NIHSS score was 3 (1–5) in 44 individuals in whom this was assessed. None of the ischaemic stroke patients had received thrombolysis prior to blood collection. Sixty-six patients who did not receive an ischaemic stroke diagnosis were also included in this study. Of these, 9 patients were diagnosed with primary haemorrhagic stroke, 26 were diagnosed with transient ischaemic attacks, and 31 were diagnosed with ischaemic stroke mimics (Fig. 1 and Supplementary Table 2). The majority of participants in the stroke mimic group were diagnosed with functional neurological disorders, migraines and/or syncope or peripheral vertigo (collectively accounting for 71% of presentations; Fig. 1).

**Table 1:** Baseline characteristics of patients included in this study

Characteristics	Whole cohort (*n* = 126)	Ischemic stroke (*n* = 60)	Non-ischaemic stroke (*n* = 66)	*P-*value
Age (years)	71.4 (60.6–79.1)	72.1 (60.7–80.5)	69.2 (60.3–77.9)	0.387
Male sex	71 (56.3%)	36 (60.0%)	35 (53.0%)	0.475
BMI	28.1 (24.9–31.0) [3]	28.6 (25.3–32.0)	27.8 (24.5–30.5) [3]	0.297
Hours from symptom onset to presentation	2.6 (1.2–4.6) [14]	2.6 (1.3–5.0) [5]	2.6 (1.1–4.3) [9]	0.481
**Smoking history**				
Never smoked	49 (38.9%)	20 (33.3%)	29 (43.9%)	0.449
Ex-smoker	54 (42.9%)	27 (45.0%)	27 (40.9%)	
Current smoker	23 (18.3%)	13 (21.7%)	10 (15.2%)	
**Comorbidities**				
Hypertension	87 (69.0%)	42 (70.0%)	45 (68.2%)	0.849
CHD	42 (33.3%)	21 (35.0%)	21 (31.8%)	0.710
TIA	42 (33.3%)	16 (26.7%)	26 (39.4%)	0.185
Stroke	51 (40.5%)	34 (56.7%)	17 (25.8%)	< 0.001
Diabetes	29 (23.0%)	15 (25.0%)	14 (21.2%)	0.675
**Prescription for**				
Aspirin	36 (28.6%)	14 (23.3%)	22 (33.3%)	0.241
Other antiplatelet drugs	10 (7.9%)	5 (8.3%)	5 (7.6%)	1.000
Anticoagulants	19 (15.1%)	8 (13.3%)	11 (16.7%)	0.628
Calcium channel blockers	27 (21.4%)	10 (16.7%)	17 (25.8%)	0.278
Beta-blockers	31 (24.6%)	13 (21.7%)	18 (27.3%)	0.537
ACE inhibitors	25 (19.8%)	14 (23.3%)	11 (16.7%)	0.379
ARBs	36 (28.6%)	14 (23.3%)	22 (33.3%)	0.241
Statins	53 (42.1%)	22 (36.7%)	31 (47.0%)	0.281
Metformin	13 (10.3%)	6 (10.0%)	7 (10.6%)	1.000
Insulin	5 (4.0%)	2 (3.3%)	3 (4.5%)	1.000

Categorical variables are presented as count and percent, and are compared between groups using the Chi-squared test. Continuous variables are presented as median and inter-quartile range and are compared using the Mann–Whitney U test. Numbers in square brackets refer to number of missing datapoints for that variable

*CHD* Coronary heart disease, *TIA* Transient ischaemic attack, *ACE* Angiotensin converting enzyme, *ARB* Angiotensin receptor blocker

Patients diagnosed with ischaemic stroke were significantly more likely to have had a prior stroke than those who received a non-ischaemic stroke diagnosis. No other differences in cardiovascular risk factors or prescribed medications were observed between the groups. Median time from symptom onset to hospital presentation for the cohort was 2.6 (inter-quartile range: 1.2–4.6) hours and did not differ between the groups.

### Primary outcome assessment: the association of serum angpt-1 with ischaemic stroke diagnosis

**Fig. 2:**
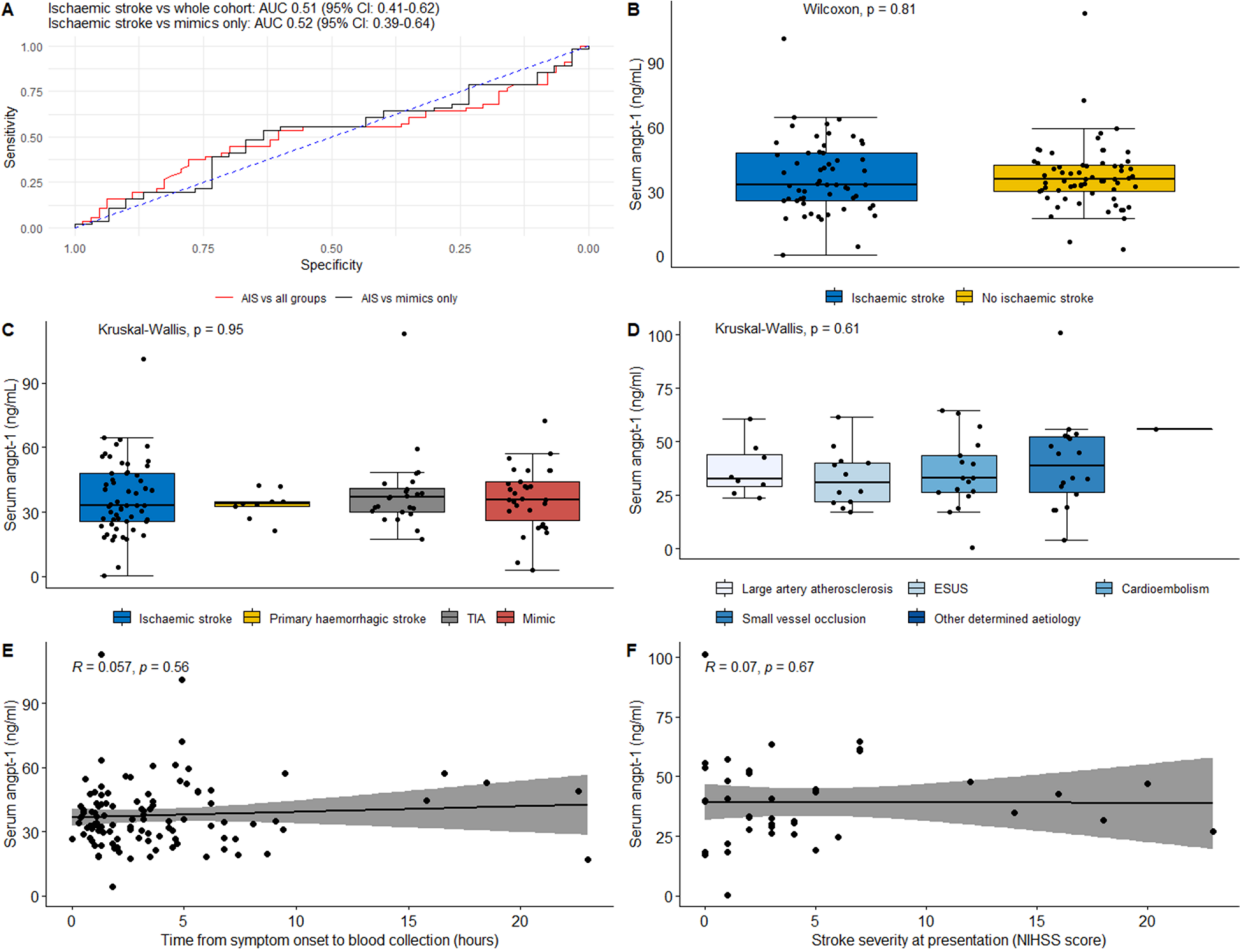
The association of serum angpt-1 with ischaemic stroke diagnosis. **A** ROC curve assessing the potential for serum angpt-1 to identify patients with ischaemic stroke when compared to the whole cohort (red line), or stroke mimics (black line). Serum concentrations of angpt-1 for patients according to stroke presence (**B**), specific presentation (**C**) or stroke sub-type according to TOAST criteria (**D**). Spearman correlations of serum angpt-1 concentration and time from symptom onset to presentation (**E**); and stroke severity as assessed by NIHSS score (**F**). ESUS: Embolic Stroke of Undetermined Source

Serum angpt-1 was detected in 119 (94.4%) of the 126 participants recruited to the study (*n* = 56 and 63 for participants who did or did not suffer ischaemic stroke, respectively; Supplement 1). ROC analysis revealed that serum angpt-1 concentration had poor ability to detect patients with ischaemic stroke in comparisons utilising the whole cohort, or in sub-analysis including patients with ischaemic stroke or stroke mimics (data from 56 participants with, and 30 without ischaemic stroke Fig. 2A and Supplement 1). Cross-sectional comparisons demonstrated no difference in serum angpt-1 concentrations between groups of patients who did or did not have ischaemic stroke (Fig. 2B and C), or between those suffering ischaemic strokes of different aetiology as determined by TOAST classification (Fig. 2D). No relationship between serum angpt-1 concentration and time from symptom onset to hospital presentation (Fig. 2E), or stroke severity as assessed by the NIHSS scale (Fig. 2F) was observed.

### Exploratory analyses: the association of serum concentrations of angiogenic proteins with ischaemic stroke

**Fig. 3:**
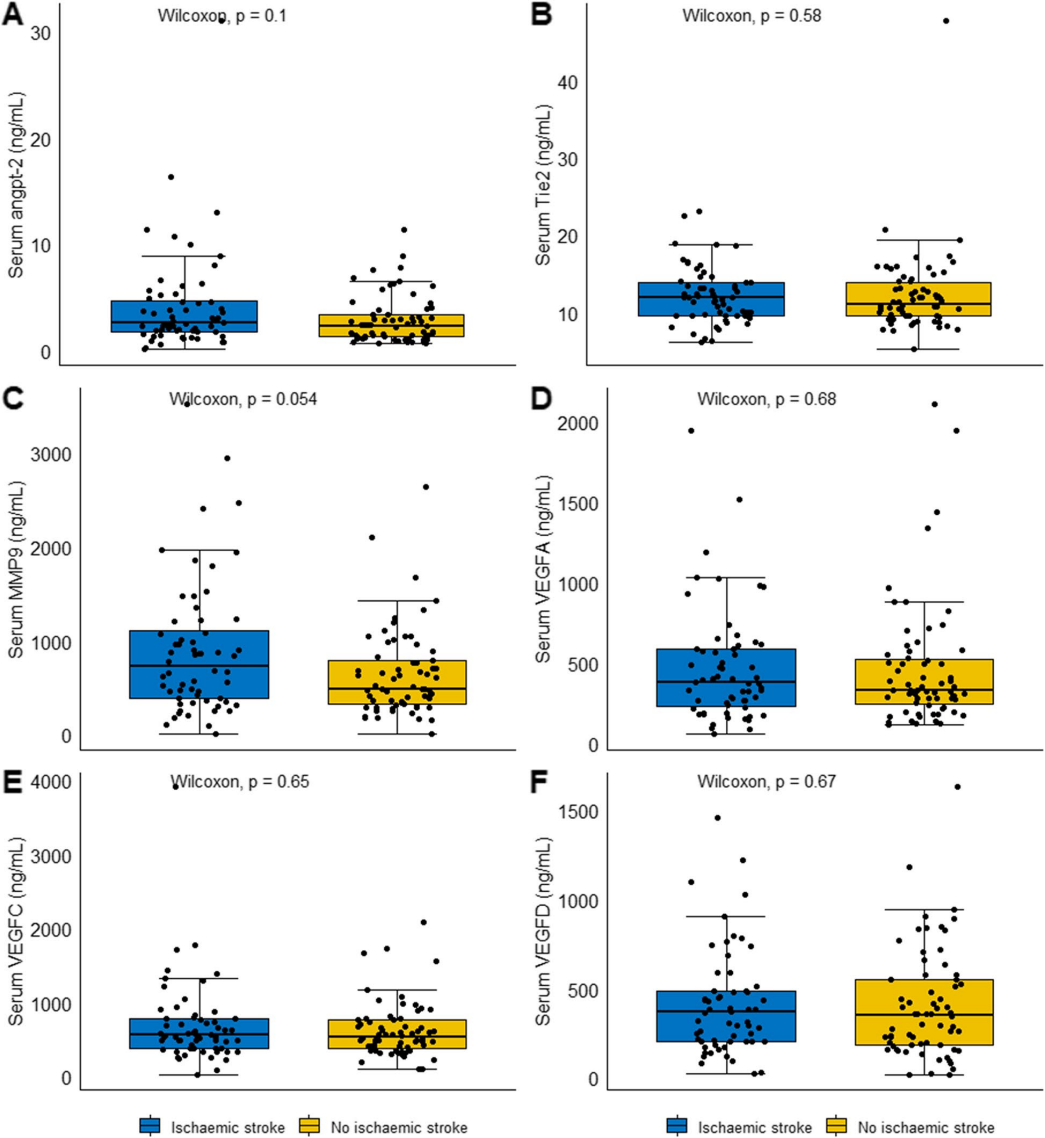
Comparisons of serum angiopoietin-2 (**A**), matrix metalloproteinase-9 (**B**), Tie-2 (**C**), VEGF-A (**D**), VEGF-C (**E**) and VEGF-D (**F**) between groups of patients who did and did not have ischaemic stroke

Correlation analyses demonstrated significant positive associations between several of the assessed proteins (Supplement 3). Cross-sectional analyses revealed no difference in serum angpt-2, Tie-2, MMP-9 or VEGF-A, -C and –D concentrations between groups of participants who did or did not have an ischaemic stroke (Fig. 3). None of the assessed serum proteins showed significant correlations with the time between symptom onset and presentation to hospital (Supplement 4). A statistically significant correlation of serum VEGF-A concentration, with NIHSS score was observed (Spearman’s rho: 0.367; *p* = 0.014; Supplement 4). Serum concentrations of the other assessed proteins were not significantly correlated with stroke severity. Cross sectional comparisons suggested significant differences in the serum concentrations of VEGF-D between presenting groups (i.e. ischaemic stroke, TIA, haemorrhagic stroke and stroke mimics), and serum angpt-2 across TOAST categories (Supplements 5 and 6). A trend suggesting potential differences in serum VEGF-D concentration across TOAST categories was observed but did not reach statistical significance (*p* = 0.055, Supplement 6). Serum concentrations of the other assessed proteins did not differ according to presentation or ischaemic stroke aetiology (Supplement 6).

## Discussion

Findings of the current study suggest that serum angpt-1 concentrations did not differ between groups of patients who did and did not have an ischaemic stroke and has poor diagnostic potential. This contrasts with findings of our previous study which reported high potential for plasma angpt-1 concentration to diagnose ischaemic stroke [14]. This difference in findings is likely reflective of the very different design of the two studies. The previous study adopted a case–control design to compare groups of patients who had an ischaemic stroke with healthy controls, whereas the current investigation consecutively recruited participants presenting for emergency assessment of stroke-like symptoms. Approximately 40% of the non-stroke patients (~ 20% of whole cohort) were diagnosed with transient ischaemic attack which may also be associated with changes in blood biomarkers. Also previous investigations have reported significant differences in the circulating concentrations of some of the investigated proteins between groups of healthy controls and individuals with health problems mimicking stroke (e.g [20]). It is also important to note that protein analysis in the current study was conducted on serum samples, compared to plasma samples in the previous report [14]. This was necessary as platelet-free plasma required by available assays could not be obtained from the current cohort. Nonetheless, previous studies have reported significant differences in serum angpt-1 concentrations between comparator groups demonstrating that serum is a suitable medium for investigating angpt-1 expression [21–23].

**Fig. 4:**
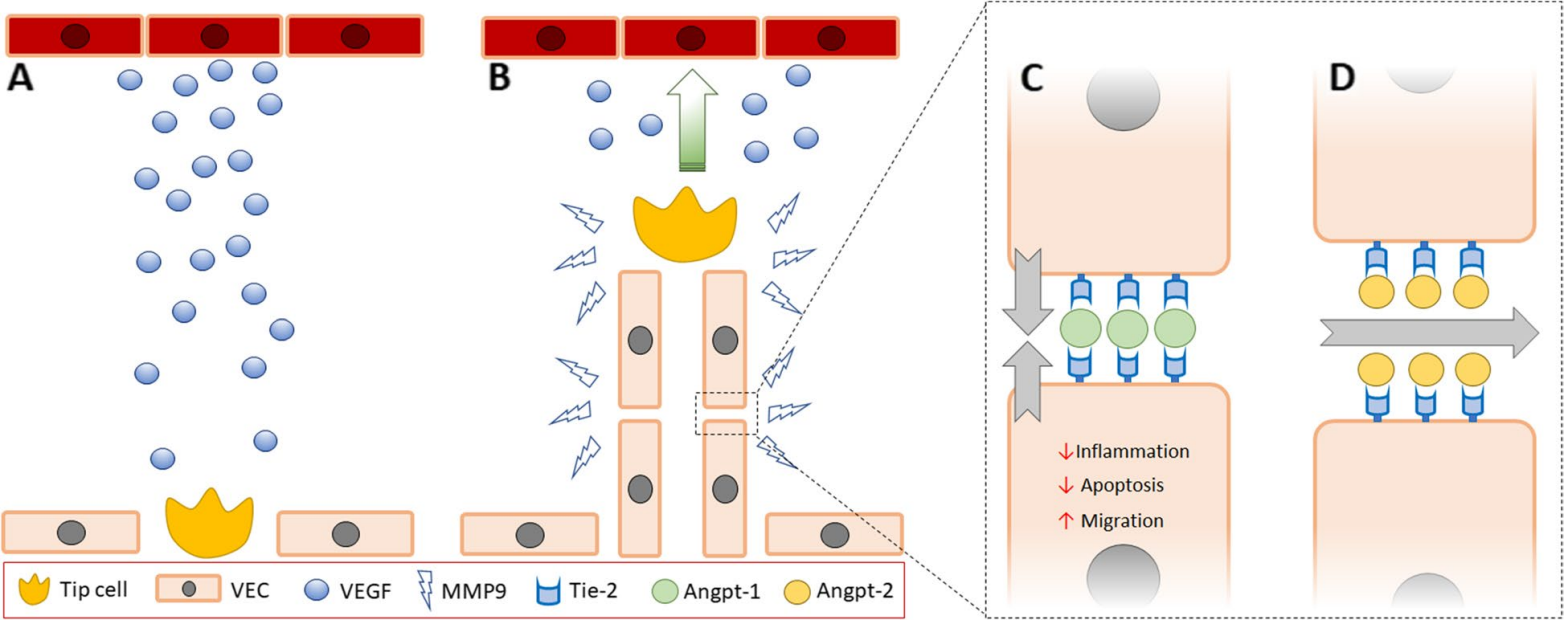
Overview of angiogenesis and rationale for biomarker selection in the current study. **A** Ischaemic insult results in the secretion of VEGF from hypoxic cells (shown in dark red). VEGF signaling stimulates the transformation of a resting vascular endothelial cell into a tip cell. **B** The tip cell guides the migration of a vascular stalk towards the area of high VEGF concentration, facilitated by the secretion of basement-degrading enzymes such as MMP-9. **C** Ligation of endothelial cell-expressed Tie-2 by angpt-1 exerts anti-apoptotic, anti-inflammatory and pro-migratory effects, and promotes tight junction formation between neighbouring cells to reduce vascular permeability. **D** Angpt-2 competes for the Tie-2 receptor to antagonise angpt-1 mediated processes

The other proteins assessed in the current study were selected due to their known involvement in angiogenesis which is tightly regulated under normal physiological circumstances, but is activated in response to ischaemic injury (Fig. 4 and references [24–28] for detailed reviews). In brief, hypoxia inducible transcription factor activation following ischaemic stroke rapidly induces the production and secretion of pro-angiogenic chemokines including VEGF from neurons, astrocytes and microglia within the ischaemic tissue [28, 29]. VEGF stimulates the differentiation of a vascular endothelial cell into a tip cell (Fig. 4A) which guides the migration of a vascular stalk towards areas of high VEGF concentration (Fig. 1B), facilitated by the release of basement-membrane degrading proteases such as MMP-9 [29]. Nascent blood vessels produced in response to VEGF are immature and highly permeable. The angpt-Tie-2 axis is the master regulator of vascular integrity [24] and available evidence suggests that processes mediated through this pathway are important in post stroke recovery. Angpt-1 is an obligate ligand for the Tie-2 receptor which is abundantly expressed throughout the vascular endothelium. Angpt-1 forms tetramers which bind to the Tie-2 receptor and exerts stabilising effects on the developing blood vessel through several mechanisms. Firstly, angpt-1/Tie-2 signalling exerts anti-inflammatory effects, and promotes endothelial cell survival, migration and proliferation (Fig. 4C and [28] for detailed review). Angpt-1/Tie-2 receptor signalling also improves the contact between adjacent endothelial cells, surrounding vascular smooth muscle cells and the basement membrane [4]. Translocation of the Tie-2 receptor to the endothelial cell boundaries also enables neighbouring cells to bind the same angpt-1 tetramer in *trans*, which contributes to the formation of tight junctions (Fig. 4C) [4, 28]. Angpt-1/Tie-2 signalling has also been suggested to confer neuro-protective effects [28, 30–32]. Observations of significantly smaller cerebral infarctions, and reduced blood–brain-barrier permeability in rodents receiving interventions to increase endogenous angpt-1 [4, 32], and better functional outcomes for patients with higher circulating angpt-1 concentrations at presentation [14], suggest that angpt-1 mediated processes are beneficial in post-stroke recovery. Angpt-2 is secreted from vascular endothelial cells in response to focal inflammation and hypoxia and competes with angpt-1 for the Tie-2 receptor [24, 27] to inhibit, or weakly stimulate Tie-2 signalling in a context-dependent manner. Elucidating of the role of angpt-2 in ischaemic stroke recovery is therefore complicated, and previous studies utilising rodent models have provided contradictory evidence of the effect of angpt-2 over expression on vascular permeability [28].

Whilst much work has been performed to understand the role of the assessed proteins in the response to ischaemia, comparatively few studies have investigated the association of these proteins with ischaemic stroke diagnosis. Meta-analyses of independent genomic studies support an association of polymorphisms of the VEGF [33] and MMP-9 [34–38] genes with increased risk of stroke. In contrast, a recent meta-analysis of case–control studies suggests no difference in total serum VEGF concentrations between groups of patients diagnosed with a stroke and healthy controls [39]. Data from the current study identified a significant difference in serum VEGF-D concentration when comparing between specific participant presenting groups (i.e. ischaemic stroke, TIA, haemorrhagic stroke or stroke mimics), however this was not upheld in primary comparisons which grouped patients based on a positive or negative ischaemic stroke diagnosis. It is therefore possible that individual VEGF isoforms may be more closely associated with specific presentations than total VEGF, and that this association may have been lost in the current study upon grouping patients according to ischaemic stroke presence or absence. Further appropriately powered studies are needed to directly assess this. Associations of blood-borne MMP-9 concentration with poor stroke outcomes have been reported across independent studies, and high circulating MMP-9 concentrations have been associated with increased risk of haemorrhagic transformation following ischaemic stroke [40, 41]; however, the association of MMP-9 with incident stroke has been less widely explored [12]. Two small studies have independently reported that circulating angpt-2 concentrations are significantly higher in patients who have experienced an ischaemic stroke, than healthy controls [42, 43], although this is contradicted by findings of the current study.

Findings of this study must be considered in line with its strengths and weaknesses. A major strength of this study was the recruitment of a clinically relevant patient population. Also, the median time from symptom onset to hospital presentation for this cohort (2.6 h) was within the 4.5 h therapeutic window for thrombolysis. Thus, the current study reflects the clinical challenge whereby an effective diagnostic must be able to discriminate patients with ischaemic stroke from a high background of non-related conditions, immediately after the event. Evidence suggests that expression of the assessed proteins increases in the peri-infarct region within hours to days of an ischaemic stroke [28]. It is therefore possible that inter-group differences in the serum protein concentrations may have been observed if patients were followed and repeatedly sampled, however, this is not suggestive of strong diagnostic potential and does not reflect clinical need. It is also important to consider that clinical notes did not include NIHSS score for all participants, and brain imaging data to estimate cerebral infarction volume was not available, thereby complicating in-depth investigation of the relationship between serum biomarker concentration and ischaemic stroke severity. Available data, however, suggest no correlation between serum angpt-1 concentration and NIHSS score. Ischaemic stroke aetiology in the current cohort was heterogeneous and we were under-powered to directly assess the relationship of circulating biomarkers with specific TOAST sub-classes, however, sample size calculations based on previous findings suggest that the study was well powered to assess the primary outcome [14]. Information on the duration of symptoms for patients diagnosed with transient ischaemic attack was also not available, meaning that potentially heterogeneous patients were grouped together. Finally, it is important to note that the study recruited participants from a single centre and further investigation in larger, independent cohorts including patients with strokes of varying severity and aetiology are therefore warranted to determine the generalisability of study findings.

In conclusion, the current study utilised serum samples collected from a clinically relevant patient cohort to demonstrate that angpt-1 has poor potential to diagnose acute ischaemic stroke. No association between ischaemic stroke diagnosis and serum concentrations of the other assessed proteins was observed.

## Supplementary Information

**Additional file 1: Supplement 1.** Number of patients in whom serum protein markers were detected. **Supplement 2.** Demographics of included participants according to presentation. **Supplement 3.** Correlation matrix showing relationship between all assessed serum proteins. **Supplement 4.** Correlation matrix showing relationship between the serum proteins with pre-hospital symptom duration and NIHSS score. **Supplement 5.** Graphs showing expression of assessed proteins for all participants according to presentation groups. **Supplement 6.** Graphs showing expression of assessed proteins for participants with ischaemic stroke according to stroke aetiology (TOAST criteria).

## Data Availability

The Queensland Research Centre for Peripheral Vascular Disease (QRCPVD) will oversee any materials sharing processes. Requests for data should be addressed to the corresponding author.
