# Supplementary material for: Serum angiopoietin-1 concentration does not distinguish patients with ischaemic stroke from those presenting to hospital with ischaemic stroke mimics
Source: BMC Cardiovasc Disord. 2022 Nov 4;22:462. doi: 10.1186/s12872-022-02918-w (PMC9636674; doi:10.1186/s12872-022-02918-w)
Supplement: Supplementary file 1 — Additional file 1: Supplement 1. Number of patients in whom serum protein markers were detected. Supplement 2. Demographics of included participants according to presentation. Supplement 3. Correlation matrix showing relationship between all assessed serum proteins. Supplement 4. Correlation matrix showing relationship between the serum proteins with pre-hospital symptom duration and NIHSS score. Supplement 5. Graphs showing expression of assessed proteins for all participants according to presentation groups. Supplement 6. Graphs showing expression of assessed proteins for participants with ischaemic stroke according to stroke aetiology (TOAST criteria). [file 12872_2022_2918_MOESM1_ESM.pdf]

Serum angiopoietin-1 concentration does not distinguish patients with ischaemic stroke from those presenting to hospital with ischaemic stroke mimics.

Joseph V Moxon PhD, Ann-Katrin Kraeuter PhD, James Phie PhD, Sheryl Juliano RN, Georgina Anderson BSc, Glenys Standley RN, Cindy Sealey BSc, Richard P White MD FRCP FRACP, Jonathan Golledge MChir FRCS FRACS

Supplementary material

Supplement 1: Number of patients in whom serum protein markers were detected

Supplement 2: Demographics of included participants according to presentation.

Supplement 3: Correlation matrix showing relationship between all assessed serum proteins

Supplement 4: Correlation matrix showing relationship between the serum proteins with pre-hospital symptom duration and NIHSS score.

Supplement 5: Graphs showing expression of assessed proteins for all participants according to presentation groups

Supplement 6: Graphs showing expression of assessed proteins for participants with ischaemic stroke according to stroke aetiology (TOAST criteria).

Supplement 1: Number of patients in whom serum protein markers were detected

Protein marker	Number of patients proteins were detected in		
	Whole Cohort (n=126)	No ischaemic stroke (n=66)	Patients with ischaemic stroke (n=60)
Angpt-1	119 [7]	63 [3]	56 [4]
	* 86 [5]	30 [1]	56 [4]
Angpt-2	126	66	60
Tie-2	126	66	60
MMP-9	125 [1]	65 [1]	60
VEGF-A	126	66	60
VEGF-C	126	66	60
VEGF-D	126	66	60

* Denotes sensitivity analysis including patients with stroke mimics only (92 participants included in total). Numbers in brackets denotes number of missing data points for that variable.

Supplement 2: Demographics of included participants according to presentation.

Characteristics	Ischemic stroke (n=60)	Primary haemorrhagic stroke (n=9)	*TIA* (n=26)	*Mimics* (n=31)	*P*-value
Age (years)	72.1 (60.7-80.5)	78.0 (71.7-87.8)	67.8 (61.6-75.0)	66.4 (57.1-74.9)	0.054
Male sex	36 (60.0%)	6 (66.7%)	15 (57.7%)	14 (45.2%)	0.543
BMI	28.6 (25.3-32.0)	27.8 (20.5-28.6)	28.7 (25.9-32.3)	27.7 (24.1-30.0) [3]	0.248
Hours from symptom onset to presentation	2.6 (1.3-5.0) [5]	3.6 (1.3-5.6) [2]	2.0 (1.0-3.2) [1]	3.2 (1.5-4.9) [6]	0.283
**Smoking history**					
Never smoked	20 (33.3%)	4 (44.4%)	13 (50.0%)	12 (38.7%)	0.594
Ex-smoker	27 (45.0%)	5 (55.6%)	8 (30.8%)	14 (45.2%)	
Current smoker	13 (21.7%)	0 (0.0%)	5 (19.2%)	5 (16.1%)	
**Cardiovascular risk factors**					
Hypertension	42 (70.0%)	8 (88.9%)	16 (61.5%)	21 (67.7%)	0.526
CHD	21 (35.0%)	2 (22.2%)	11 (42.3%)	8 (25.8%)	0.570
TIA	16 (26.7%)	1 (11.1%)	18 (69.2%)	7 (22.6%)	<0.001
Stroke	34 (56.7%)	4 (44.4%)	4 (15.4%)	9 (29.0%)	0.001
Diabetes	15 (25.0%)	3 (33.3)	5 (19.2%)	6 (19.4%)	0.765
**Prescriptions for**					
Aspirin	14 (23.3%)	3 (33.3%)	11 (42.3%)	8 (25.8%)	0.332
Other antiplatelet drugs	5 (8.3%)	0 (0.0%)	2 (7.7%)	3 (9.7%)	1.000
Anticoagulants	8 (13.3%)	3 (3.3%)	3 (11.5%)	5 (16.1%)	0.424
Calcium channel blockers	10 (16.7%)	3 (3.3%)	6 (23.1%)	8 (25.8%)	0.526
Beta-blockers	13 (21.7%)	3 (3.3%)	6 (23.1%)	9 (29.0%)	0.746
ACE inhibitors	14 (23.3%)	2 (2.2%)	5 (19.2%)	4 (12.9%)	0.689
ARBs	14 (23.3%)	2 (2.2%)	10 (38.5%)	10 (32.3%)	0.478
Statins	22 (36.7%)	5 (55.6%)	16 (61.5%)	10 (32.3%)	0.084
Metformin	6 (10.0%)	2 (2.2%)	1 (3.8%)	4 (12.9%)	0.351
Insulin	2 (3.3%)	0 (0.0%)	1 (3.8%)	2 (6.5%)	0.887

Quantitative data are presented as median and inter-quartile range. Numbers in square brackets relate to number of missing data-points for that variable. P-value relates to the comparison of all groups (Kruskal-Wallis test for continuous variables or Fisher’s exact test for nominal variables).

Supplement 3: Correlation matrix showing the relationships between the assessed serum proteins

	Angpt-1	Angpt-2	Tie-2	MMP-9	VEGF-A	VEGF-C	VEGF-D
**Angpt-1**							
**Angpt-2**	-0.131<br>(p=0.157)						
**Tie-2**	0.152<br>(p=0.100)	0.173<br>(p=0.053)					
**MMP-9**	0.239<br>(p=0.009)	0.087<br>(p=0.336)	0.246<br>(p=0.006)				
**VEGF-A**	0.254<br>(p=0.005)	0.189<br>(p=0.034)	0.129<br>(p=0.151)	0.319<br>(p<0.001)			
**VEGF-C**	-0.009<br>(p=0.921)	0.051<br>(p=0.569)	0.087<br>(p=0.330)	0.130<br>(p=0.148)	0.022<br>(p=0.810)		
**VEGF-D**	-0.024<br>(p=0.795)	0.336<br>(p<0.001)	-0.035<br>(p=0.696)	0.039<br>(p=0.669)	-0.003<br>(p=0.974)	0.115<br>(p=0.198)	

Shown are Spearman correlation coefficients (top row of cells) and calculated p-values (bottom row of cells).

Supplement 4: The association of serum protein concentrations with pre-hospital symptom duration and stroke severity (NIHSS score).

Serum marker	Assessed in whole cohort		Assessed in patients with ischaemic stroke	
	Time to presentation		NIHSS score	
	Spearman's R	p-value	Spearman's R	p-value
Angpt-2	-0.116	0.224	0.258	0.091
Tie-2	0.014	0.882	-0.219	0.154
MMP-9	0.031	0.747	0.216	0.158
VEGF-A	0.006	0.948	0.367	0.014
VEGF-C	-0.094	0.325	-0.294	0.053
VEGF-D	-0.058	0.543	0.161	0.297

*P-value relates to comparison across patients grouped by TOAST classification (Kruskal-Wallis test).

Supplement 5: Graphs showing expression of assessed proteins for all participants according to presentation groups

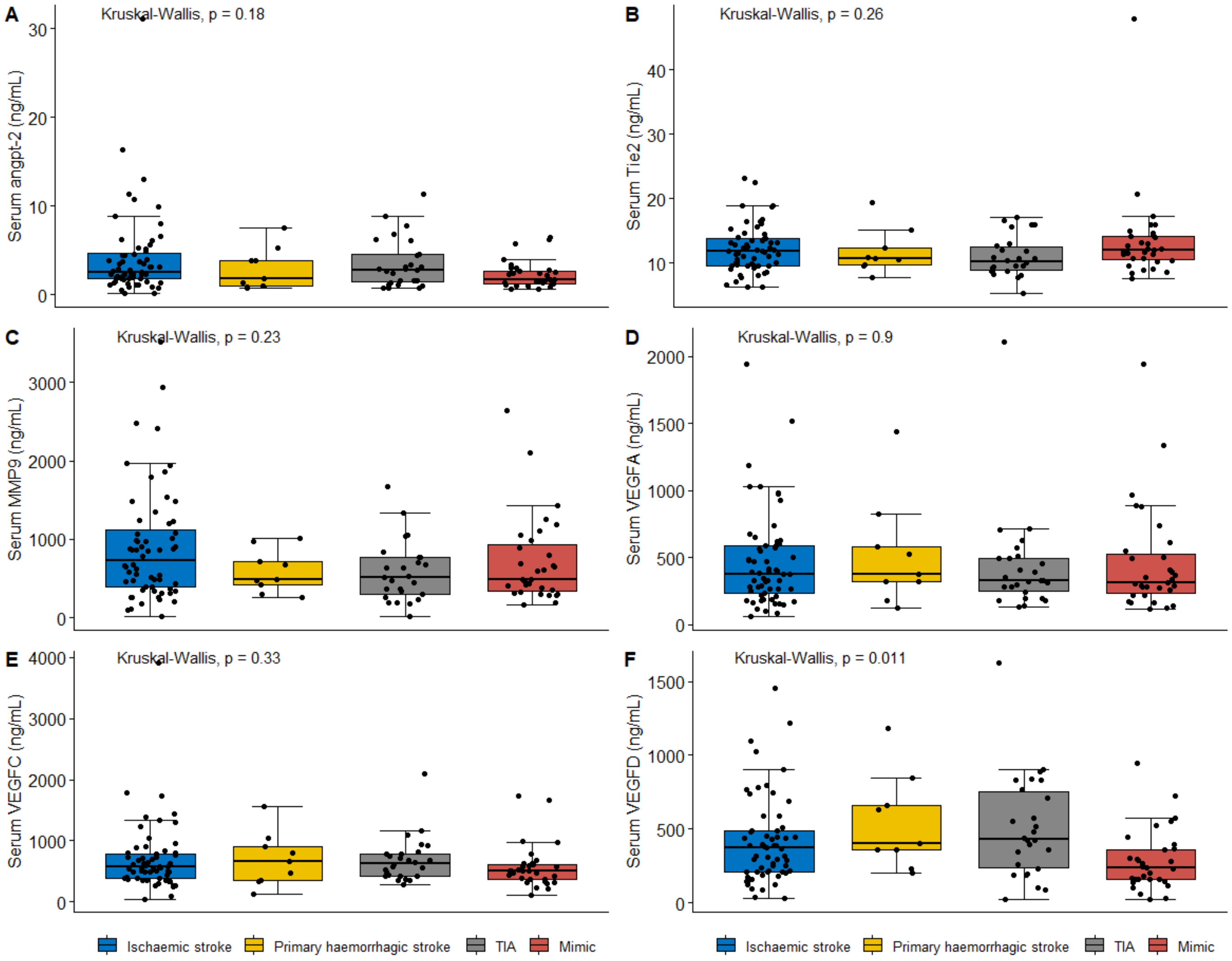

Supplement 6: Graphs showing expression of assessed proteins for participants with ischaemic stroke according to stroke aetiology (TOAST criteria).

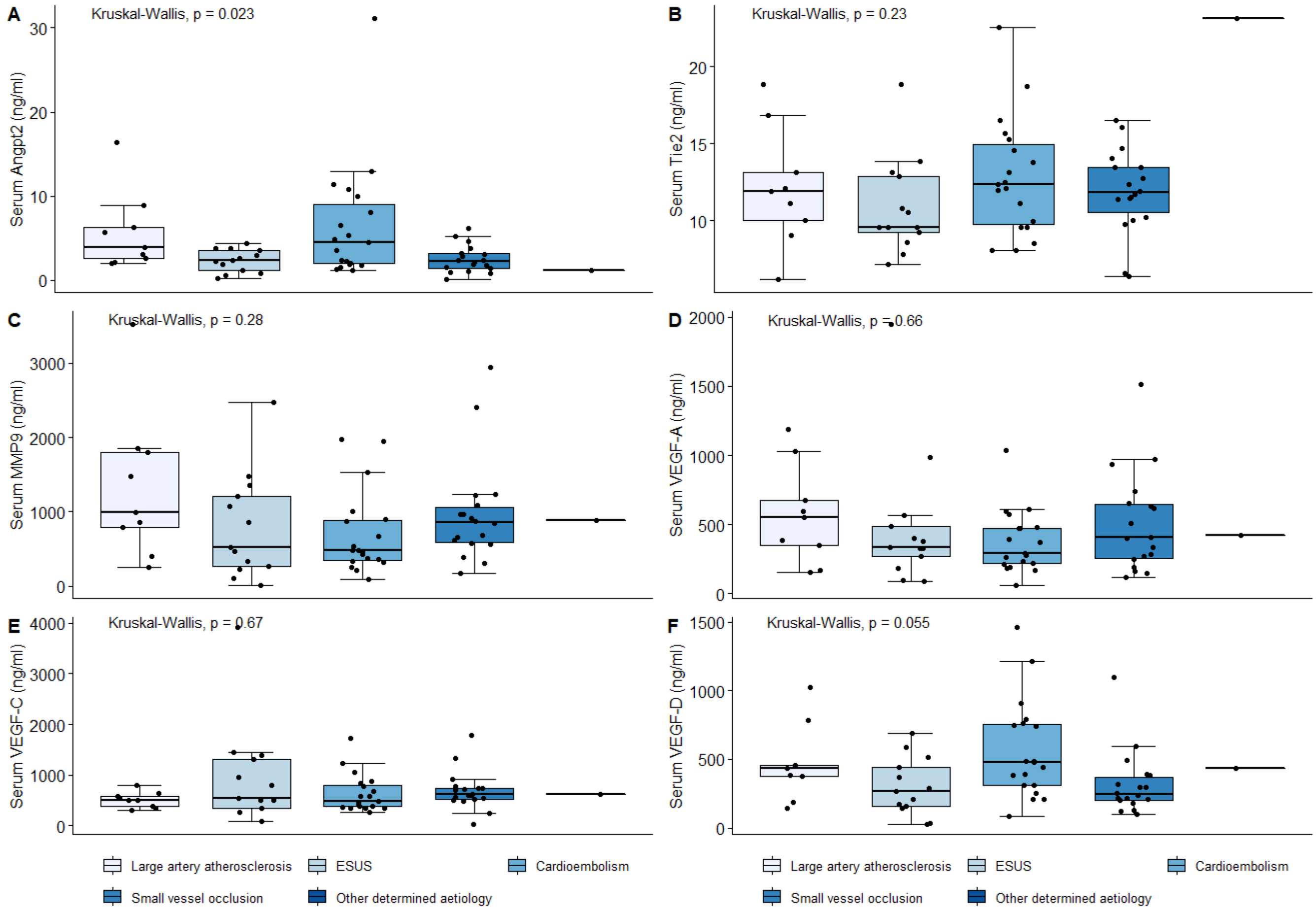

ESUS: Embolic stroke of unknown source
